# Experience in prenatal genetic testing and reproductive decision-making for monogenic disorders from a single tertiary care genetics clinic in a low-middle income country

**DOI:** 10.1186/s12884-023-05698-z

**Published:** 2023-06-10

**Authors:** Amna Hanif, Fizza Akbar, Salman Kirmani, Amyna Jaffarali, Ghulam Zainab, Ayesha Malik, Zeeshan Ansar, Bushra Afroze

**Affiliations:** 1 Department of Paediatrics & Child Health, Aga Khan University (AKU) Hospital, Stadium Road, Karachi, Pakistan; 2 Department of Obstetrics & Gynaecology, Aga Khan University (AKU) Hospital, Karachi, Pakistan; 3 Section of Molecular Pathology, Department of Pathology and Laboratory Medicine, Aga Khan University (AKU) Hospital, Karachi, Pakistan

**Keywords:** Genetic testing, Informed reproductive decision making, LMICs, Prenatal diagnosis, Rare disease burden

## Abstract

**Objectives:**

Explore health-care seeking behaviour among couples with pregnancies at-risk of monogenic disorders and compare time duration for obtaining Prenatal Genetic Test (PGT) results based on (i) amniocentesis and Chorionic Villus Sampling (CVS) (ii) in-house testing and out-sourced testing. Report the spectrum of monogenic disorders in our cohort.

**Methods:**

Medical records of women consulting prenatal genetic counselling clinic at Aga Khan University Hospital, Karachi from December-2015 to March-2021 with history of miscarriage or a monogenic disorder in previous children were reviewed.

**Results:**

Forty-three pregnancies in 40 couples were evaluated, 37(93%) were consanguineous. Twenty-five (63%) couples consulted before and 15(37%) after conception. Thirty-one (71%) pregnancies underwent CVS at the mean gestational age of 13-weeks and 6-days ± 1-week and 3-days and amniocentesis at 16-weeks and 2-days ± 1-week and 4-days. PGT for 30 (70%) pregnancies was outsourced. The mean number of days for in-house PGT was 16.92 ± 7.80 days whereas for outsourced was 25.45 ± 7.7 days. Mean duration from procedure to PGT result was 20.55 days after CVS compared to 28.75 days after amniocentesis. Eight (18%) fetuses were homozygous for disease-causing variant for whom couples opted for termination of pregnancy (TOP). Twenty-six monogenetic disorders were identified in 40 families.

**Conclusion:**

Proactive health-care seeking behaviour and TOP acceptance is present amongst couples who have experienced a genetic disorder.

## Introduction

Prenatal genetic diagnostic testing confirms the genetic disease in the fetus and is useful for perinatal decision-making and management. Genetic diseases are a major cause of morbidity and mortality worldwide and a great burden on society. Countries in South Asia are known for their endemic genetic diseases and are commonly described as ‘a hotspot for hemoglobinopathies’ [1]. According to the global report on birth defects, the estimated prevalence of all genetic birth defects is 74.3 per 1,000 live births in Pakistan [2], which is mainly attributable to the cultural practice of consanguineous marriages. According to the Pakistan Demographics and Health Survey (2012–2013) half of all marriages in Pakistan are consanguineous [1, 3].

Given the genetic disease burden in Pakistan and to reduce their prevalence, genetic testing strategies need to be devised along with better genetic counseling services that are delivered in an empathetic and non-directive manner to empower families to make autonomous decisions, consonant with their religious and ethical beliefs and circumstances [2].

Initially, prenatal genetic testing (PGT) was confined to the diagnosis of chromosomal anomalies such as Down syndrome (Trisomy 21) with conventional tests such as karyotyping. However, with the recent advances in genetic testing and diagnostic breakthroughs, a gamut of genetic disorders can now be detected [4]. Conventional tests have now been replaced by Next Generation Sequencing that has opened avenues in providing accurate diagnoses for genetic diseases in families at risk. Offering prenatal diagnosis for known familial variants, after earlier confirmed genetic disease diagnosis in a family or after identification of disease carrier status of the parents, is now a standard clinical practice. Furthermore, depending on the diagnostic status, now prenatal whole exome and whole genome sequencing may also be offered [5, 6].

In this study, we analyse a series of 43 pregnancies in 40 couples in which monogenic disorders were diagnosed within our institution and outsourced laboratories abroad. This study provides important insights into the prenatal health care seeking behaviour among couples with pregnancies at-risk of being affected by monogenic disorders and their attitudes towards reproductive decision making in a resource limited country with deep religious roots. This work also shows the spectrum of monogenic disorders seen in the Prenatal Genetics Clinic (PGC) at our centre.

## Methods

This is a single centre retrospective review of medical records of mothers who consulted the Prenatal Genetics Clinic (PGC) in the Department of Paediatrics and Child Health at Aga Khan University Hospital (AKUH), Karachi, Pakistan between December 2015 and March 2021 with a history of miscarriage, recurrent neonatal death or a monogenic disorder in previous children Due to high rate of consanguinity; monogenic autosomal recessive disorders are common in our population. Many of the monogenic disorders can manifest in the fetal life resulting in a miscarriage, therefore couples who had undergone one or more miscarriages were also included in our study.

The data from this work between January 2016 to July 2018 has been previously reported by S. Munim et al., from our centre [7].

Medical records were reviewed, and data was collected from mothers’ charts and stored on a pre-structured questionnaire, after receiving study approval and informed consent waiver from Ethical Review Committee (ERC) of The Aga Khan University Karachi (ERC # 2021–5961-16,778).

Details of demographic and laboratory data including age of the couple at presentation and consanguinity, pre-conception visit, clinical presentation and available phenotypic characteristics or diagnostic studies of previously affected child were recorded.

Both pre- and post-test counselling were done by the medical geneticists at the PGC. The Fetal-Maternal medicine team (FMMT) performed chorionic villus sampling (CVS) or amniocentesis to obtain fetal cells from placental tissue or amniotic fluid, respectively. To minimize maternal cell contamination (MCC), an experienced specialist performed the procedure. Fetal DNA was extracted from the fetal cells and sent along with samples of the parents’ DNA sample, the latter of which was used as a control. At our institution 16 short tandem repeat (STR) kit for MCC was used to check the informative markers. The minimum informative markers as cut-off were set to be more than two in fetal DNA for diagnosis of significant MCC [8]. Whereas, for samples that were outsourced, MCC of fetal specimens were tested using the out-sourced laboratory’s DNA Genotyping Panel [9].

Genetic testing for the diagnosis of the Delta F508 variant in *CFTR* for cystic fibrosis (CF), spinal muscular atrophy (SMA), Duchene muscular dystrophy (DMD) and beta thalassemia was available at our institution. Testing for other conditions was performed at various reference laboratories in the US and the UK.

Results of prenatal genetic diagnostic tests and the parents’ decision regarding continuation of pregnancy were also recorded. Date of procedure (CVS or amniocentesis) and fetal DNA extraction and the time taken for the fetal DNA to reach the overseas laboratory were all recorded. Time duration to obtain fetal genotype results after CVS versus amniocentesis and for in-house versus outsourced PGT were compared. We used t-test to compare the mean of the two groups and considered a probability of p < 0.05 as statistically significant.

## Results

Out of all couples who presented to PGC, 40 couples qualified and chose to proceed with invasive prenatal testing over the study period. Three of these 40 couples consulted us during two pregnancies, and 10 out of these 43 pregnancy cases, had been previously reported [7]. A large proportion of our cohort was consanguineous; 37(92.5%). The median age of the mothers was 29 ± 7.25 years, and that of the fathers was 33 ± 6.5 years. Twenty-five (63%) married couples consulted the medical geneticist before planning their pregnancy whereas 15(37%) presented after conception. All the 40 couples had a family history of a previously affected child, fetus or relative, out of whom 9(22.5%) couples did not have a molecular diagnosis for previously affected children and underwent carrier testing that was informative for offering them prenatal testing. Thirty-one(77.5%) couples had a definitive genetic diagnosis of previously affected child, fetus or relative based on laboratory investigations.

**Fig. 1:**
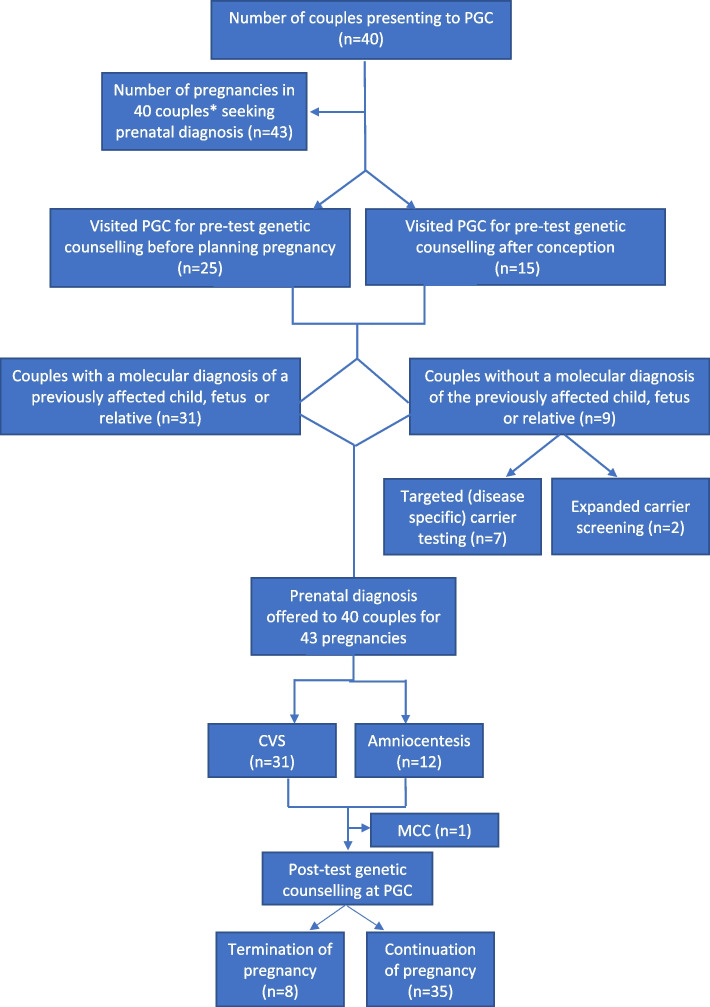
describes a framework used for couples presenting to PGC requiring PND for monogenic disorders

**Fig. 2:**
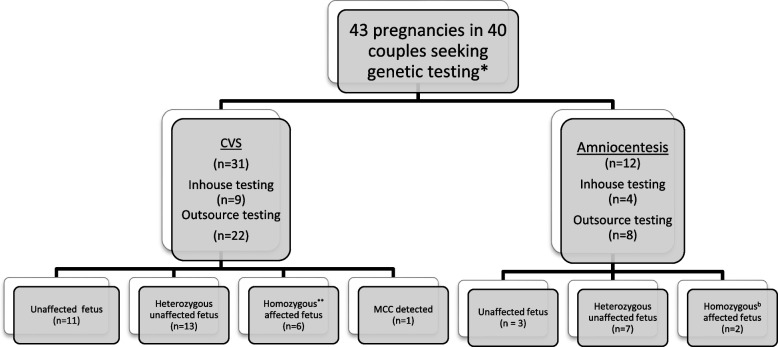
describes the outcome of prenatal genetic testing

Out of the 40 couples, in two consanguineous couples, there was no affected child or fetus, however there was a family history and confirmed molecular diagnosis of a monogenic disease in second degree relatives (nephews/nieces) of either of the partners. For these two couples, prenatal testing was offered for familial variants only after the carrier status of both partners was confirmed. Figure 1 describes the framework used for couples presenting to PGC requiring PND for monogenic disorders. Figure 2 presents the outcome of genetic diagnostic testing results for couples visiting the Prenatal Genetics Clinic.

The mean gestational age at prenatal visit was 67 ± 23 days. In a total of 43 procedures, none resulted in a complication of miscarriage. However, in two women, undergoing CVS there was a dry tap which was followed by a successful amniocentesis. Successful CVS and amniocentesis were considered while reporting the study results. Thirty-one (72%) pregnancies underwent testing by chorionic villus sampling (CVS) and the remaining 12(28%) by amniocentesis. The mean gestational age at the time of CVS was 97 ± 10 days (13 weeks and six days ± 1 week and three days) whereas that at amniocentesis was 114 ± 11 days (16 weeks and 2 days ± 1 week and four days). The overall mean gestational age at testing was 102 days (14 weeks and four days ± 1 week and five days).

PGT for 30(70%) of the 43 pregnancies were outsourced to reference laboratories in the US and the UK. For the remaining 13(30%) pregnancies PGT was available at our institution. The overall mean number of days for the prenatal genetic results to be available after the procedure was 23.0 ± 8.0 days. The mean number of days for in-house PGT was 17.08 ± 6.44 days whereas for outsourced PGT was 25.45 ± 7.78 days, which was statistically significant(p = 0.001).

Overall, the mean duration of testing from procedure to prenatal-genetic results was 20.5 ± 7.63 days after CVS compared to 28.75 ± 7.03 days after amniocentesis, which was statistically significant(*p* = 0.001). The mean duration for fetal DNA extraction from a CVS sample followed by MCC detection through polymerase chain reaction (PCR) took an average of 7.91 ± 4.73 days compared to an amniocentesis sample that took 17.25 ± 6.20 days. This difference was statistically significant(*p* = 0.001). Table 1 provides a comparison of the mean duration of results for the different parameters of testing.

**Table 1:**
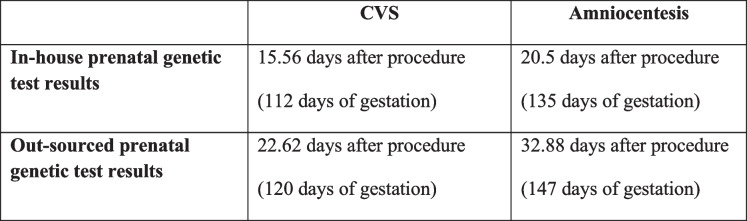
Comparison of the mean duration of results for different parameters of testing

In eight pregnancies (19%), the fetus was homozygous for a disease-causing variant and in all these pregnancies the couples opted for TOP. The rest of the couples continued with their pregnancy. One of the fetal genetic tests; did not yield a result due to 100% MCC detection. Testing was carried out for 27 monogenic disorders in our cohort, of which 26 were autosomal recessive, while one was an X-linked disorder, excluding the sample with 100% MCC.

The sample with MCC had undergone CVS at 12 weeks of gestation and the sample was outsourced. The sample was received at the overseas lab at 15 weeks and by the 16 weeks of gestational age, the lab informed us about the 100% MCC in the sample. After this, the couple was counselled regarding the option of amniocentesis as well as the possibility of complications of miscarriage and probable logistical bottleneck of not receiving the result in time to proceed with TOP the fetus is affected, and the result is received after the 24^th^ week of gestation which is the institutional TOP cut-off.

In nine pregnancies (21%), the prenatal diagnosis was sought for SMA. Spectrum of disorders for which prenatal genetic diagnosis was offered and the pregnancy outcomes for each family are summarised in Table 2. In one pregnancy, prenatal diagnosis was sought for two monogenic disorders, beta-thalassemia and *LAMA2* related muscular dystrophy.

**Table 2:**
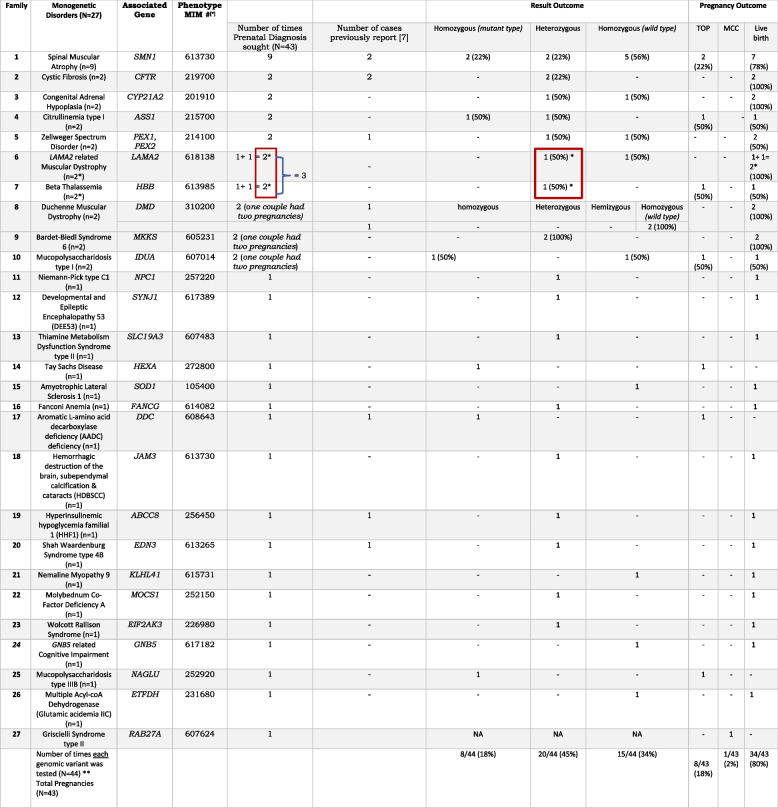
Is inserted in a separate document as it required “Landscape Orientation”

## Discussion

This study reports a formal experience of our genetics team involved in prenatal genetic diagnostic testing and risk counselling for a cohort of couples presenting with history of monogenic disorders at a tertiary care setting in Pakistan.

PGT identifies life limiting disorders for which neonatal outcomes can be optimized and more importantly the option of TOP could be provided [10, 11]. One of the authors reported that 22% of the couples opted for voluntary TOP in the absence of prenatal diagnosis for the fear of having another child with a genetic disorder [12]. This is contrary to the common belief where some segments of our population do not terminate a pregnancy involving an anomalous child due to ethical concerns, restraining religious or cultural beliefs in an Islamic country like Pakistan. In such settings, it becomes pivotal to provide accurate justification for TOP in a timely manner. In this study all the eight couples with an affected fetus opted for TOP. In Pakistan, majority of jurists from different Schools of Islamic Jurisprudence have concluded based on development of fetal organs that legal TOP is permitted before 120 days of gestation counted from date of conception (134 days or 19 weeks from last menstrual period) [13]. However, the cut-off for TOP at our institution is 168 days (24 weeks), based on the age of fetal viability [14]. According to Sect. 338 of Pakistan Penal code, the legal abortions are permitted before 120 days of gestation to either save a woman’s life or to provide “necessary treatment” [15]. However, the term “necessary treatment” is not defined in law and is open for interpretation. In Pakistan, the discordance between law on TOP and practice places responsibility on doctors to determine conditions for which TOP should be offered [16]. Lack of well-defined legislation leaves the doctors and institutes in ethical and legal dilemmas. Therefore, it is important for the healthcare providers to layout guidelines for the cut-off of TOP and further expound on the term “necessary treatment”. It will then be worthwhile to debate on national level involving the policy makers and accordingly revisit the code regarding the cut-off for TOP through more concerted efforts involving medical, religious, and legal experts.

Genetic diagnostic testing has always required considerable time and it gets further challenging if the couple presents during pregnancy. We observed that results of fetal genetic testing require 25 days when outsourced, comparing with 17 days with in-house testing; necessitating that investing in personnel and infrastructure development for in-house testing is crucial. Our study also demonstrated that CVS is preferred over amniocentesis, from the time of sample collection to sample preparation and obtaining the genetic test result. Subsequently, the mean duration of testing from procedure to results was 20.5 days after CVS compared to 28.75 days after amniocentesis. This delay poses a substantial time difference for a gestational timeline and decision making.

However, it was noted, that gestational age at which CVS is being performed at our institution is later, reported at 13 weeks and six days (± 1 week and three days) than the international standards, ranging between 10–13 weeks [17]. Thus, this necessitates institutional strategizing and advanced training of FMMT for optimizing the time of procedure performed. In accordance with international standard, if it is performed at average; on 12^th^ week of gestation (10–13 weeks), then test results that take 20.5(± 7.63) days may become available by 16^th^ week of gestation, decreasing the delay by two weeks, approximately.

However, CVS has a greater overall risk of miscarriage (around 2%, versus 0.5–1% for amniocentesis) [18, 19] as well as maternal cell contamination (MCC) (< 5%) [20]. Out of the 43 samples, one sample obtained for genetic testing via CVS was found to have MCC. This could be because it is difficult to thoroughly remove maternal decidua from fetal cells in a CVS sample [21, 21]. In our work, following MCC detection, fetal genetic testing was not possible as the results could reflect maternal genotype rather than fetal genotype. Unavailability of back-up fetal culture facilities limits the option of fetal prenatal testing in case of MCC detection, which is an important counselling point.

Given the high burden of genetic diseases in our country as evidenced, and the need to expedite the diagnosis and decision making for TOP, it is crucial to develop local testing [23]. As the cost of healthcare is out-of-pocket, a family who has a child with a genetic disorder faces immense financial and emotional challenges. Therefore, devising strategies and creating awareness for the prevention of such inherited diseases becomes crucial.

63% of these couples with a history of an affected pregnancy or child had visited the PGC before conception. This shows an increase in awareness and demand among the population when compared to a previous study in 2007 in the same settings where 48% of the couples had visited pre-conception [13].

However, it was observed that no couple in this study cohort sough pre-marital genetic counselling. This contrasts with Iran where 80% of the clients seek pre-marital counselling because of consanguinity. [24, 24]. In this context, many Muslim countries have implemented laws mandating a pre-marital screening for the entire population before they obtain a marriage certificate [25].

This study also highlights the importance of performing population screening for common genetic conditions like SMA in addition to beta thalassemia. In Pakistan about 5250 infants with beta thalassemia are born annually [26]. The exact prevalence of SMA in our population is still not known but as evident by this study and previous work from our institution, it is seen in relatively high numbers in the Genetics and Fetal Medicine clinics [7]. As observed, a fifth of our study cohort sought prenatal testing for SMA. Possible explanation for this skewing could be: (i) a higher disease burden of SMA in our population due to high rates of consanguineous marriages, (ii) paediatricians’ trained to identify the clinical phenotype of SMA, based on electromyography and nerve conduction studies and (iii) live births and availability of cost-effective in-house testing for molecular diagnosis confirmation. While these factors are required to be further explored, a combination of all these factors is likely playing a role in identifying a prominent percentage of SMA cases, as also reported in other consanguineous populations [26].

## Limitations

The study sample was diverse with regards ethnicity since couples from all over the country visit our institution. However, due to the limited sample size, it is not entirely representative of the Pakistani population and hence not generalizable. As AKUH is a private quaternary medical centre, where patients pay out-of-pocket, the cost of the prenatal testing including procedure and testing especially when outsourced is also a limiting factor for several families.

In this study the relationship between the socioeconomic status and the influence on reproductive decision-making was not systematically assessed. Therefore, more demographic information on the couples seeking PGT e.g. educational levels, socio-economic status need to be objectively recorded through standardized questionnaires in future. Educational level of the couple could be an important factor in their understanding and behaviour for seeking PGT and accepting TOP. It may be inferred that since most of the testing samples were outsourced, the patients with a higher socioeconomic status could afford it.

The mean gestational age was 13 weeks and six days (± 1 week and three days) at with CVS was performed at our institution, with is much later then the recommended international standard. This is likely a result of expertise and comfort level of our FMMT; with regards to the preferred time to perform CVS. This is a contributing factor in the delay of receiving a prenatal diagnosis, that needs to be strategized by advancing the training and updating the practice of performing the invasive prenatal procedure at optimal time, as recommended by international clinical practice organizations such as The American College of Obstetricians and Gynaecologists.

## Conclusion

Our data from the PGC staffed by medical geneticists, shows that proactive health care seeking behaviour in the country is present amongst couples who have cared for a child with the medical needs of a genetic disorder or have had recurrent miscarriages in the past. Given the life limiting nature and financial challenges imposed by genetic disorders, couples make autonomous decisions about TOP if the fetus is affected. Our work proposes a framework, and describes challenges and possible solutions for establishing a prenatal genetics service, that can be replicated in other medical centres in Pakistan and other LMICs, with formally trained genetics healthcare providers. Subsequently, we also show that there is a need for expanding in-house testing capacity, as well as advanced training of FMMT to perform CVS as per international standards. In our reported experience, cultured CVS sampling is preferred over direct amniocentesis; that reduces the test turnaround time from 25 to 17 days for in-house prenatal testing and from 29 to 20 days for outsourced prenatal testing, respectively. As observed, a diverse range of diseases in a relatively small cohort goes to show that not only population-wide carrier frequency information for monogenetic disorders is needed to elucidate the rare disease burden at a national level, but country-wide formal PGCs, counselling and diagnostic services are direly needed.

## Data Availability

The data that supports the findings of this study are available from the corresponding author upon reasonable request.

## Abbreviations

CF: Cystic Fibrosis
CVS: Chorionic Villus Sampling
DMD: Duchenne Muscular Dystrophy
FMMT: Fetal-Maternal Medicine Team
LMIC: Low Middle-Income Country
MCC: Maternal Cell Contamination
PCR: Polymerase Chain Reaction
PGC: Prenatal Genetics Clinic
PGT: Prenatal Genetic Test
SMA: Spinal Muscular Atrophy
STR: Short Tandem Repeats
TOP: Termination of Pregnancy
